# *Padina boergesenii* mediated synthesis of Se-ZnO bimetallic nanoparticles for effective anticancer activity

**DOI:** 10.3389/fmicb.2024.1358467

**Published:** 2024-02-26

**Authors:** Balaji Thirupathi, Yimtar Lanutoshi Pongen, Govindarajan Rasiravathanahalli Kaveriyappan, Pavan Kumar Dara, Suresh Rathinasamy, Saranya Vinayagam, Thanigaivel Sundaram, Baek Kwang Hyun, Thirumurugan Durairaj, Suresh Kumar Rajamani Sekar

**Affiliations:** ^1^Department of Biotechnology, Faculty of Science and Humanities, SRM Institute of Science and Technology, Kattankulathur, India; ^2^Department of Biotechnology, Yeungnam University, Gyeongsan, Gyeongbuk, Republic of Korea; ^3^Research and Development Centre, Greensmed Labs, Thoraipakkam, Chennai, Tamil Nadu, India; ^4^Department of Biosciences, Saveetha School of Engineering, Saveetha Institute of Medical and Technical Sciences, Chennai, India; ^5^College of Natural Sciences, SNNPR, Arba Minch University, Arba Minch, Ethiopia

**Keywords:** *Padina boergesenii*, green synthesis, Se-ZnO nanoparticles, anticancer, hepatoblastoma cell lines, MCF7 breast cancer cell line

## Abstract

**Introduction:**

Evaluating the anticancer property of Padina boergesenii mediated bimetallic nanoparticles.

**Methods:**

The present study focuses on synthesizing Se-ZnO bimetallic nanoparticles from an aqueous algal extract of brown algae *Padina boergesenii*.Synthesized Se-ZnO NPs were characterized by UV, FTIR, SEM-EDS and HRTEM for confirmation along with the anticancer activity by MTT assay.

**Results:**

The UV gave an absorbance peak at 342 and 370 nm, and the FTIR showed functional groups involved in synthesizing Se-ZnO NPs. The TEM micrographs indicated the crystalline nature and confirmed the size of the Se-ZnO NPs to be at an average size of 26.14 nm. Anticancer efficacy against the MCF-7 breast and HepG2 (hepatoblastoma) cell lines were also demonstrated, attaining an IC_50_ value of 67.9 µg and 74.9 µg/ml respectively, which caused 50% cell death.

**Discussion:**

This work aims to highlight an effective method for delivering bioactive compounds extracted from brown algae and emphasize its future therapeutic prospects. The potential of Selenium-Zinc oxide nanoparticles is of great interest due to the biocompatibility and low toxicity aspects of selenium combined with the cost-effectiveness and sustainability of zinc metal. The presence of bioactive compounds contributed to the stability of the nanoparticles and acted as capping properties.

## 1 Introduction

The field of material science and nanotechnology are constantly introducing new and improved techniques for the fabricating stable metallic nanoparticles, these fabricated nanoparticles have uses in chemical, pharmacological, medical, and several different industrial fields (Mohamed et al., 2019). Nanoparticles are diverse and include organic, inorganic, ceramic, and carbon-based metallic oxide particles. Here, organic nanoparticles are nontoxic and biodegradable, while inorganic nanoparticles are more stable but hydrophilic (Ijaz et al., 2020), generally metal precursors are used to synthesize metal nanoparticles, which are more efficient and reactive. Initially, nanoparticles were produced through the deformation of macroparticles or small particles by top-bottom and bottom-up approaches (Berta et al., 2021). This is attributed to the self-assembly of particles in physical and chemically consumable ways (Mukherjee et al., 2021), which were proven to be expensive and toxic to the environment (Hendi et al., 2023). An alternative method is green chemistry, where different metal precursor solutions are combined with the inclusion of appropriate reducing agents (organic extract) to instigate the structural changes of metallic nanoparticles (Makada et al., 2023); called green synthesis, which is apparently more cost-effective, reliable, safe, and nontoxic to the environment (Ijaz et al., 2020). According to researchers who have performed the synthesis of bimetallic nanoparticles (Merugu et al., 2021), it is primarily the binding of two metals to improve and give a synergistic effect to the performance and activity of the nanoparticles, exhibiting both the characteristics of the bonded metals (Hosny et al., 2022). Unique combinations of bimetallic particles are key for their optical, magnetic, and photothermal properties, which are very effective in bio-imaging and drug delivery for diseases (Cheng et al., 2021). Bimetallic nanoparticles (BNPs) are important in biomedical applications due to their safety, stability, and low toxicity (Behera et al., 2020). BNPs are generally reduced from metal ions to particles by biomolecules (flavonoids, phenols, tannins, terpenoids, vitamins, and alkaloids), contributing to their biocompatibility (Berta et al., 2021). The novel characteristics of the BNPs, coupled with the bioactive compounds synergistically indicate to be an effective method in improving the therapeutic applications and possibilities for drug delivery.

Cancer, a significant health challenge with the aging population, lifestyle variables, environmental alterations, genetic and hereditary factors contribute to its significant prevalence (Imran et al., 2017). To combat this nanosized formulations of designed medication treatments can potentially treat cancer by targeting the multiplying malignant cells eliminating the need for current available therapies which are harmful plus generally redundant, affecting the viability of non-cancerous cells as well (Jain et al., 2021). The use of nanoparticles in its many different forms employ a possibility to mitigate the side effects incurred with traditional cancer treatment. The study conducted and highlighted below might not be a solution but rather a key insight into the developmental phase for the fight against the cancer. Combining different metals to enhance the efficacy as well as the characteristics of the combined drug. Thorough our literature study, Se-NPs have shown a novel potential to cause the intracellular ROS reaction against ovarian cancer cells (A2780) that induces apoptosis due to the upregulation of P53 and Bax genes (Amiri et al., 2021). The same also induces DNA damage in HepG2 cells, resulting in cell cycle arrest, which inhibited the proliferation of cancer cells (Li et al., 2018 Fouda et al., 2022). Similarly for the case of ZnO NPs synthesized by employing plant material as a reducing and capping agent provided characteristics such as simple availability, environmentally friendly, and affordability (Umamaheswari et al., 2021). In a study by Sathappan et al. (2021), ZnO NPs using an aqueous stem extract of *Cissus quadrangularis* yielded spherical, wurtzite-shaped NPs with higher anticancer activity against pancreatic adenocarcinoma cells. The bimetallic NPs are also shown to have enzymatic like activities in addition to the feasibility in drug delivery (Niu et al., 2022); enzymatic cascade utilizing palladium and platinum nano-reactor was able to target overexpressed CD44 in target tumor cells as well as apt decomposition after reaction (Ming et al., 2020). Similarly plant extract capped AuPtNPs were shown to have great efficacy with minimum toxicity in conjunction with Doxorubicin (Oladipo et al., 2020).

From this investigation and basing on the results of some recent studies, which found that the synthesized mono-metallic particles, i.e., Zinc and selenium, themselves have anticancer potential and can be easily fabricated using green synthesis techniques (Sathappan et al., 2021; Umamaheswari et al., 2021; Hariharan et al., 2023), we designed a study to combine the biocompatibility of Se-NPs and the cost-effectiveness of ZnO-NPs to increase the efficacy of delivering our extract’s potential bioactive molecules for treating breast and liver cancer cell lines. The study involves the intricate fabrication of a bimetallic nanoparticle with both the Selenium as well as Zinc metal synthesized with our extract, i.e., *Padina boergesenii* against MCF7 breast cancer and HepG2 liver cancer cell lines as well as to test its bioactive potency against the same.

## 2 Materials and methods

### 2.1 Chemicals

The standard reagents, including selenium selenite and zinc acetate, were procured from the SRL chemicals. The MCF-7 cell line was sourced from NCCS, Pune and was maintained through the propagation of stock cells in a DMEM medium supplemented with 10% fetal bovine serum (FBS), penicillin (100 IU/mL), and streptomycin (100 μg/mL). The cells were then cultured in a controlled environment at 37°C with 5% CO_2_ to ensure optimal growth conditions.

### 2.2 Sample collection and seaweed extraction

The Seaweed (*Padina boergesenii*) was collected from the Mandapam coastal region of southern

Tamil Nadu, Rameswaram, India (Algotiml et al., 2022). The samples were transported in polythene bags and cleaned with fresh water, removing the impurities. The samples were then cleaned with a brush, as described by Algotiml et al. (2022) and Thanigaivel et al. (2019). To remove the epiphytes and dust, distilled water was used. After washing, the biomass was shade-dried for about 2 weeks, powdered using a mixer grinder, and stored for further experiments. 5 grams of powdered sample was soaked in 100 mL of distilled water and kept in a water bath and sonicator at 50°C for 2 h. Following the protocols of Mirzaei et al. (2021), the sample was filtered with Whatman filter No.1 paper thrice and the filtrate was stored at 4^o^C.

### 2.3 Synthesis of selenium and zinc oxide nanoparticles

The selenium-zinc oxide NPs were synthesized using a slightly modified technique described by Mirzaei et al. (2021) and Dlamini et al. (2023), where 30 mL of an aqueous solution of seaweed extract was transferred into a 250-ml Erlenmeyer flask, 10 mL of zinc acetate (5 mM), and 10 mL of sodium selenite (5 mM) were added to the flask (Mirzaei et al., 2021). After adding the metal precursors, it was kept in the magnetic stirrer for 48 h at normal room temperature. The change in color and precipitation indicated the synthesis of bimetallic nanoparticles (Liang et al., 2022). The NP-containing solution was centrifuged at 14,000 rpm for 20 min. at a temperature of 4°C and washed with distilled water three times by centrifugation to remove the impurities from the sediment pellet. The collected pellet was kept overnight in an air oven at 50°C (Balaji et al., 2023).

#### 2.3.1 Characterization

The synthesized bimetallic NPs (Se-ZnO NPs) were characterized using different analytical techniques. The aqueous extract and synthesized NP solutions were analyzed using a UV–visible spectrophotometer (SHIMADZU, UV 3600 PLUS Model) at 200–800 nm range (Thanigaivel et al. 2022). A Bruker-XRD X-ray diffractometer was used to analyze the crystallinity of the synthesized NPs; diffract degree (10° to 100° theta degree). The attributed functional groups of active metabolites that constitute the synthesis of NPs were studied by FTIR analysis (SHIMADZU, IRTRACER 100 Model). The morphological and elemental mapping was carried out using HR-SEM (Hi-Resolution Scanning Electron Microscope) and HR-TEM (High-resolution Transmission Electron Microscope) (JEOL, Japan). The DLS and Zeta potential analyzers were used to analyze the particle size and stability of the sample (Malvern).

### 2.4 Rheology

The rheological properties (Shear Stress vs. Shear Rate and Viscosity vs. Shear Rate) of Se-ZnO NPs and Se-ZnO NPs + SWE (see weed extract) were measured according to the following method as described by Dara et al. (2020); Dara et al. (2021), using Brookfield DV-III Ultra TM Programmable Rheometer (Brookfield Engineering Laboratories INC, Middleboro, USA). The measuring geometry used was 5 cm cone and plate spindle (CP- 41 model) with a gap of 0.05 mm and a shear rate ranging from 1 to 200 s^−1^. A flow curve was obtained by plotting shear stress vs. shear rate and viscosity vs. shear rate values using a steady-state flow program. Herschel-Bulkley Model was selected as the best-fit model based on standard error. The Herschel-Bulkley Law model equation,Ʈ = Ʈ∘ + kD^η^ Where, Ʈ is the shear stress (Pa), Ʈ_o_ is the yield stress (Pa), D is the shear rate (s-1), k is the consistency index, and ƞ is the flow index (dimensionless). The consistency index (k) and flow behavior index (n) of Se-ZnO NPs and Se-ZnO NPs + SWE were determined by the steady-state flow program software.

### 2.5 *In vitro* anti-cancer activity

MCF-7 breast cancer and HePG2 liver cancer cell lines were purchased from NCCS Pune, India. The cell lines were maintained in a DMEM medium containing FBS (Fetal Bovine Serum), Penicillin (100 IU/mL), and streptomycin (100 μg/mL) and incubated in 5% CO_2_ at 37°C in a humid atmosphere condition. The cells in the T-25 culture flask were trypsinized when they reached 90% capacity; this was determined using a hemocytometer. After which, the trypsinized cells were adjusted to 1.0 × 10^5^ cells/mL on the respective media containing 10% FBS. The cell suspension media was added to the 96-well plate and incubated for 24 h in 5% CO_2_, after which monolayer cells were observed to be attached to the bottom of the plate. The DMEM media was removed, and the wells were washed with PBS. One hundred microliter of different concentrations of test samples were added to the partial monolayer in the microtiter plates and incubated at 37°C for 24 h in a 5% CO_2_ atmosphere. Following the incubation period, the test solutions within the wells were discarded, and 20 mL of MTT solution (2 mg/mL in PBS) was introduced to each well. Subsequently, the plate was placed in an environment with 5% CO_2_ at 37°C for 4 h. To dissolve the MTT and generate insoluble formazan crystals, the supernatant was aspirated, and 100 μL of DMSO was dispensed into each well. An aliquot of 100 μL of test samples at different concentrations (6.25, 12.5, 25, 50 and 100 μg/mL) was added to the partial monolayer and incubated at 37°C for 24 h in a 5% CO_2_ atmosphere. The drugs 5-FU (5-fluorouracil) and Doxorubicin were used as standard for MCF-7 breast cancer and HePG2 liver cancer cell lines, respectively. The plates were gently agitated for several minutes to facilitate dissolution followed by measuring OD at 570 nm. The cell viability was determined by applying the following formula and expressed in percentage.


$$\mathrm{Viability}\;\left(\%\right)=\frac{\mathrm{Sample}\;\mathrm{Abs}}{\mathrm{Control}\;\mathrm{ABs}}\times 100$$

## 3 Result and discussion

### 3.1 Synthesis of the selenium and zinc oxide nanoparticles

In the green synthesis of nanoparticles, plant extracts are used as secondary metabolites in a safe, environmentally friendly manner. Due to the association between plant material and metal precursors, this procedure reduces metal precursors to unique nanoparticles with unique properties (Navada et al., 2023). The nucleophilic character of phenols and flavonoids and their affinity for metal ions make them essential components and chelating agents for synthesizing Au-NPs, as mentioned by Raghunandan et al. (2010). The compounds that serve as chelating agents can either be organic or inorganic. A chelate is composed of two or more ligands that can have covalent bonds, coordinate linkages, or be bidentate or multidentate, which can form complex chelates when they bind to metal ions^+^ (Flora and Pachauri, 2010; Ahmad et al., 2019).

Bhuyar et al. (2020) study shows that *Padina* sp. contains bioactive metabolites such as polyphenols and carboxylic acids, which are avail in the extract and are responsible for the synthesis of the silver NPs. Selenium (Se) is a typical nutrient that can be combined with zinc oxide nanoparticles (ZnO) and Se–ZnO NPs show higher antibacterial activity than pure ZnO NPs. The addition of Se may also enhance optimization and the electronic structure as well as surface properties (Song et al., 2022). The selenium and zinc metal precursors are reduced by the aqueous seaweed extract of *P. boergesenii*. The color change occurs in the mixture after 48 h, then it adjusts the pH value to 7.0 with 1 N of the NaOH solution. The extract of *P. boergesenii* acts as a capping agent and biosurfactant to chemically reduce the selenium-zinc metal ions to form metal nanoparticles. Polyphenols in aqueous seaweed extracts can reduce the macro molecules into nanoparticles, with the presence of reducing agents like sugars, polysaccharides, peptides, and pigments in the *Padina* sp. (Bhuyar et al., 2020; Mahmood Ansari et al., 2021). The bimetallic nanoparticles were successfully synthesized from the algal-based extract of *G. corticata* and are used to treat the antioxidant, antibiofilm, and anticancer studies demonstrated by Mirzaei et al. (2021). An assessment of the anticancer activity of the synthesized bimetallic nanoparticles against breast cancer (MCF-7) and liver cancer (HepG-2) was conducted in this study.

### 3.2 Ultraviolet–visible spectrophotometry analysis

**Figure 1:**
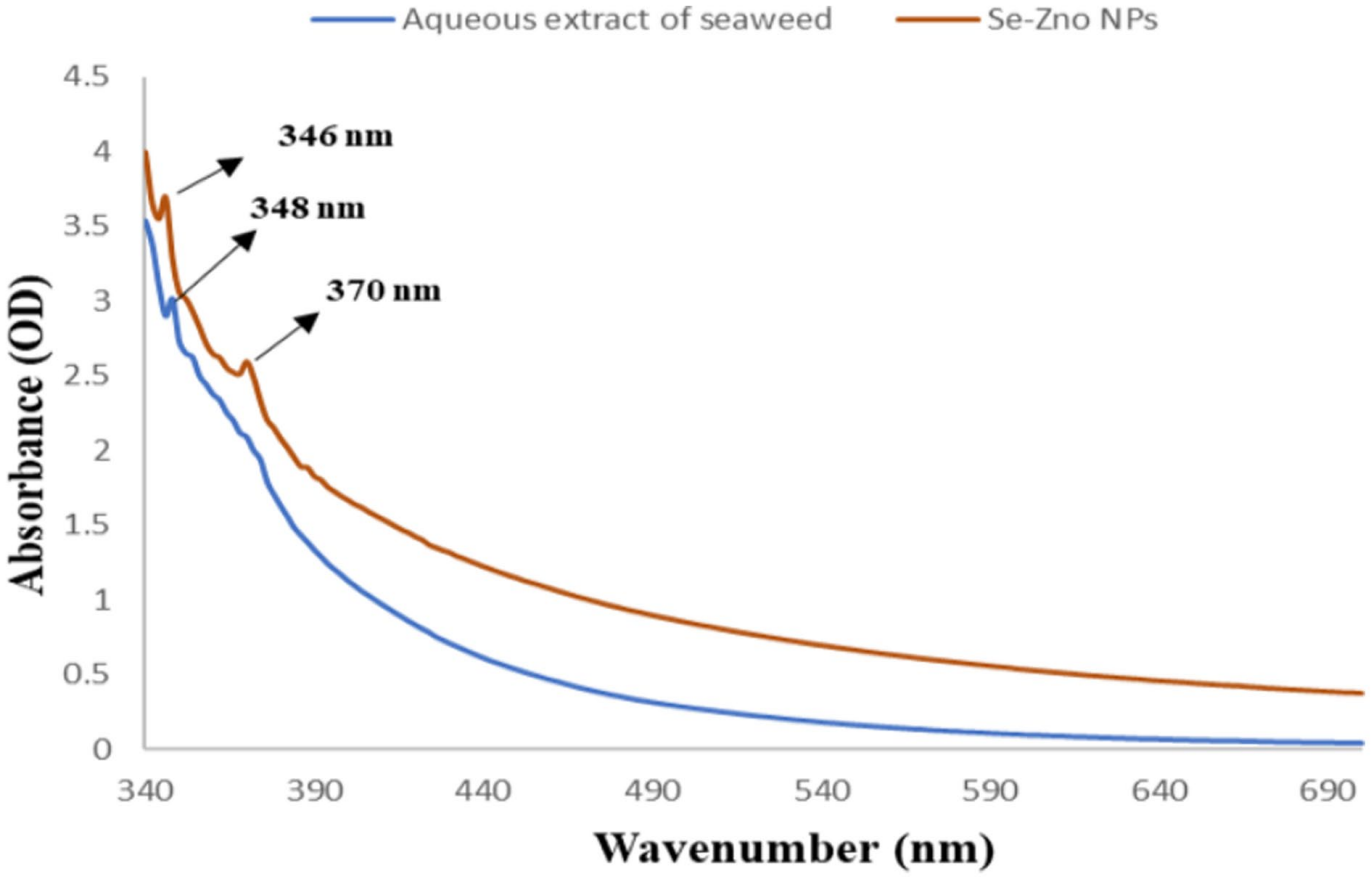
UV–visible spectrophotometer analysis of both aqueous extract of *P. boergesenii* and synthesized bimetallic nanoparticles.

A significant color change was observed during the process of synthesis of the nanoparticles, from a slight yellowish color of the extract to a dark brown attribute. This visually confirmed the formation of the nanoparticles. We believe that the change in color was an indication of the reduction of metal ions to metal NPs after the 48-h incubation and constant stirring (Mirzaei et al., 2021). This result indicates a polydispersity of metallic NPs based on the slightly broad SPR peak at 342 and 370 nm presented in Figure 1. Likewise, few studies have reported the synthesis of selenium NPs using a variety of reductants (Cittrarasu et al., 2021). The study found a strong absorbance peak at 342 nm in spectroscopic analysis for ZnO nanoparticles, as mentioned in the study by Srujana and Deepa (2022). The peak through the UV-spectrophotometer was observed to be between 342 and 370 nm; the range between 300 to 400 nm regions denotes the synthesis of the bimetallic nanoparticles in this specific range of wavelength as observed in other studies (Mirzaei et al., 2021). The addition of both metal precursors reacted with the seaweed extract, resulting in a change in color from pale yellow to dark brown, which may be due to the excitation of the surface plasmon vibrations responsible for synthesizing the nanoparticles. This was further confirmed by UV–visible analysis (Ghosh et al., 2015; Mirzaei et al., 2021; Hosny et al., 2022).

### 3.3 Fourier transform infrared spectroscopy analysis

**Figure 2:**
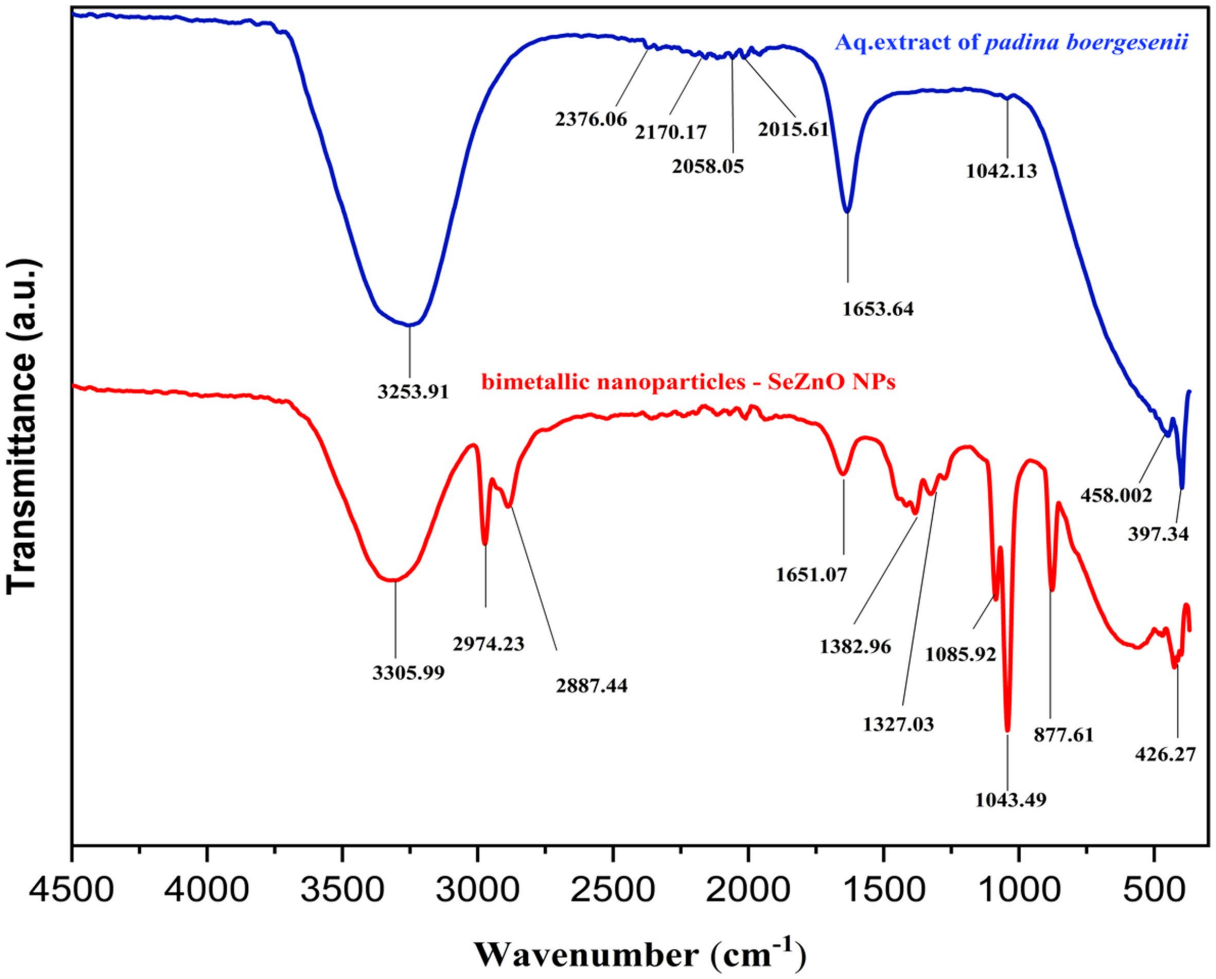
The FTIR spectra for the synthesized bimetallic nanoparticls (Se-ZnO NPs).

The FTIR spectrum (Figure 2) shows the stretching and vibrations from the aqueous extract of seaweed acting as a reducing agent to make a metal nanoparticle from the metal ions (Merugu et al., 2021). The seaweed extract shows the obtained peaks mentioned in the Figure 2. The peaks were observed at 3523, 2376, 2,170, 2058, 2015, 1,653 and 1,042. There were a number of functional groups associated with these bands, including alcohols, Phenols(O-H), carbon dioxide(O=C=O), thiocyanate(S − C ≡ N), isothiocyanate (−N=C=S), alkene and sulfoxide R − S(=O) respectively. These are functional groups is present in the aqueous extract of the *Padina boergesenii* (Mickymaray, 2019; Kalasariya et al., 2023). The peak was obtained for Se-ZnO NPs at 3305 cm^−1^ attributed to the O-H (alcohols and phenols) stretching vibrations (Saif et al., 2016; Demir et al., 2018). The peaks at 2974 cm^−1^ and 2,887 cm^−1^ are associated with hydrocarbon (C-H) vibrations of alkene (Mane et al., 2021; Hossain et al., 2023). The peak at 1,651 cm^−1^ corresponds to -C=O and C=C stretching vibrations (Torres-Liminana et al., 2022). The absorption bands at 1,382 cm^−1^ and 1,327 cm^−1^ are associated with C-N stretching of aromatic amine groups and N-O symmetric stretching of nitro compounds, respectively (Rastogi and Arunachalam, 2012; Nagalingam et al., 2018). The peaks observed at 1085 cm^−1^ correspond with the C-O of the alcoholic group (Aroob et al., 2023). The band at 1,043 cm^−1^ relates to the C-N amines. The selenium and zinc metal oxygen appeared at 426.27 cm-1 (Jyoti et al., 2016). As shown in Figure 2, all of these functional groups are present in the spectra of bimetallic NPs, nevertheless, the pre-shift and post-shift in peaks might be due to the interactions between Se and ZnO nanoparticles, as mentioned in the previous study of Hosny et al. (2022). It may be concluded that stretches and vibrations of functional groups such as phenolic compounds, aromatic amine groups, nitro compounds, and aliphatic amines were key for the synthesis of the bimetallic nanoparticles. The phytochemicals present are believed to behave as reducing as well as stabilizing agents for the Bi-NP formation.

### 3.4 X-ray diffraction crystallographic analysis

**Figure 3:**
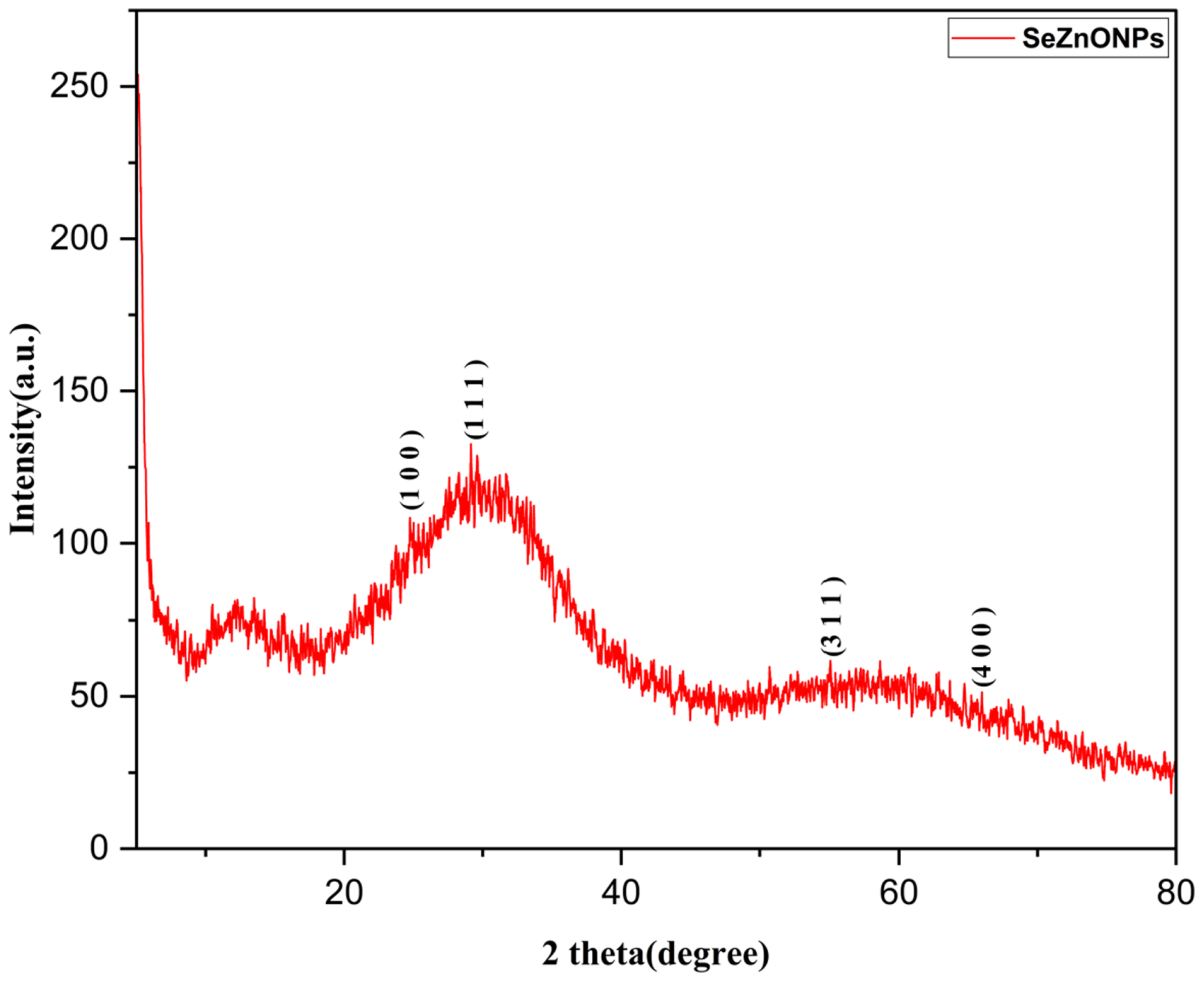
X-ray diffraction (XRD) pattern for bimetallic nanoparticles (Se-ZnO NPs).

XRD analysis is an effective method for estimating material structure, crystal size, and crystallinity in organic, inorganic, and metal oxide materials (Menazea et al., 2021). The XRD diffraction spectrum of the synthesized bimetallic nanoparticles (Se-ZnO NPs) is shown Figure 3. A clear and sharp Bragg reflection was not observed in Figure 3. According to the pattern, the green synthesized nanoparticles were slightly crystalline nature, which also agrees with the literature, indicating their nature of the structure. The amorphous nature of Se-ZnO bimetallic nanoparticles can be attributed to a complex interplay between surface effects, chemical composition and thermal stability, which can all influence nanoparticle structural properties. Since, the peaks were observed in our study as follows 23.8^o^, 29.07^o^, 53.3^o^ and 67.9^o^ responsible for the planes of (1 0 0), (1 1 1), (3 1 1), and (4 0 0), the same has been compared with the previous studies (Taha et al., 2019; Mirzaei et al., 2021). In addition, the ZnO nanoparticle peaks were confirmed the presence of bi-metallic patterns at this range 54, 66 and 69 and same have been discussed in the Hashem et al. (2023). The X-ray diffraction (XRD) peaks of the Se-doped nanoparticles (NPs) exhibit widening, and reduced intensity compared to the pristine ZnO NPs (Taha et al., 2019). The amorphous peaks were observed in the XRD pattern. If it has to achieve crystalline form, Se-ZnO NPs need to be heated to a temperature of approximately 400°C. At this temperature, it can degrade the bioactive metabolites of the sample and stop the function of biological activities, as discussed by Indhira et al. (2023). Consequently, this indicates that the nanomaterials are crystalline and that phytochemicals serve as stabilizing agents.

### 3.5 Scanning electron microscope

**Figure 4:**
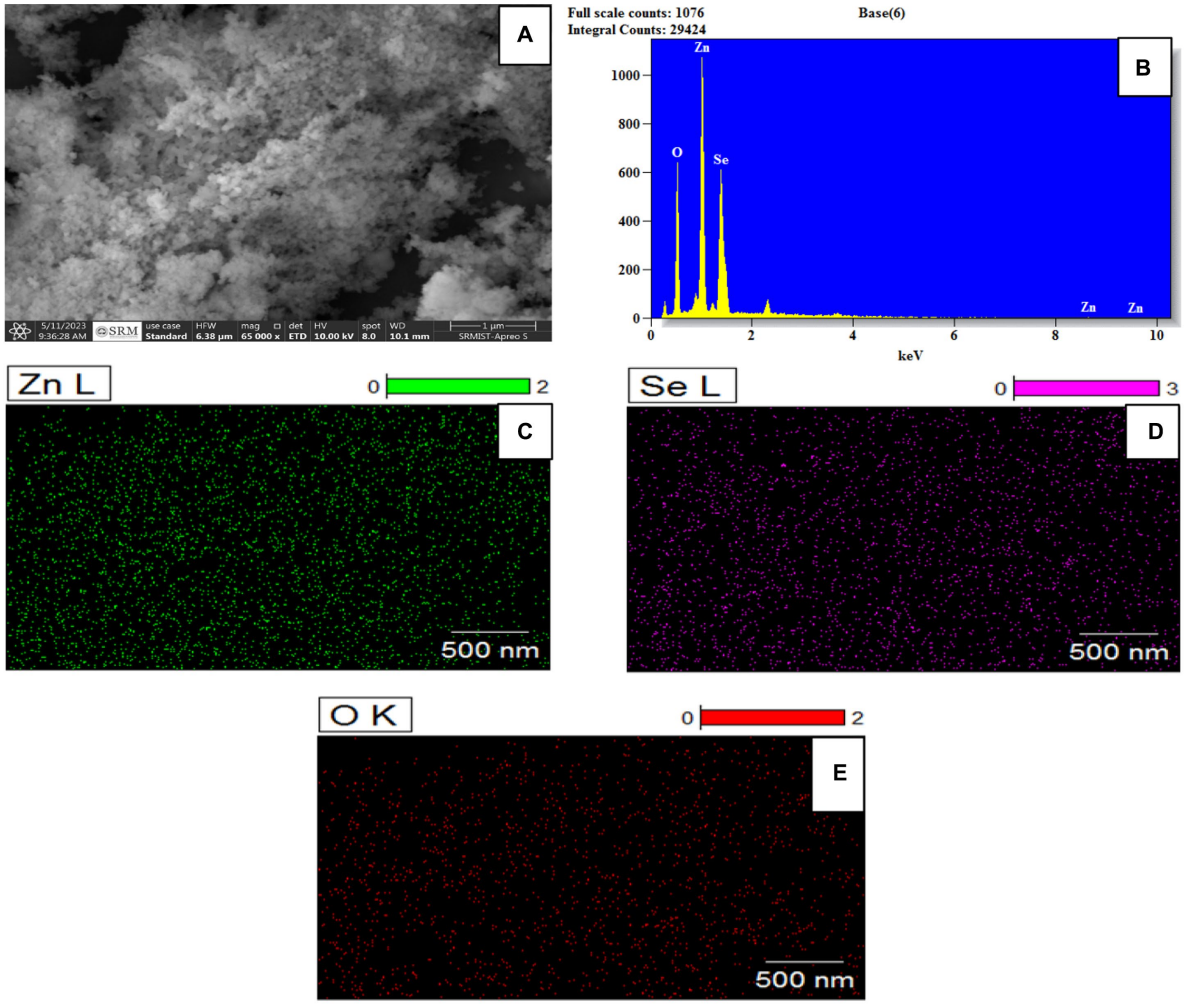
**(A)** HRSEM image **(B)** EDS spectrum and **(C–E)** Elemental color mapping of bimetallic nanoparticles.

The morphological and topographical surfaces established by using the HR SEM for nanoparticles are shown in Figures 4A,B HRSEM was used for a magnification of 12,000 and a scale of 500 nm (Chand et al., 2020). That captured nanoparticle image shows the agglomeration of the particles, which is nearly spherical in shape as mentioned in Figure 4A. EDX analysis helpful to estimate the composition of chemicals were shown in Figure 4B. The sharp peak was contributed for the Zinc, and medium and small peaks represented corresponding for the Selenium and ZnO NPs as mentioned by Cai et al. (2022). The unwanted impurity peaks appeared during the washing process. The bimetallic nanoparticles (Se-ZnO NPs) directly implicated in the elemental mapping for distinguishing the elements, illustrated elements Zn (bright color; green), Se (bright color; pink), and oxygen (bright color; red), were confirmed by EDS mapping images shown in Figures 4C–E likewise, the study of Awed et al. (2021).

### 3.6 Transmission electron microscope

**Figure 5:**
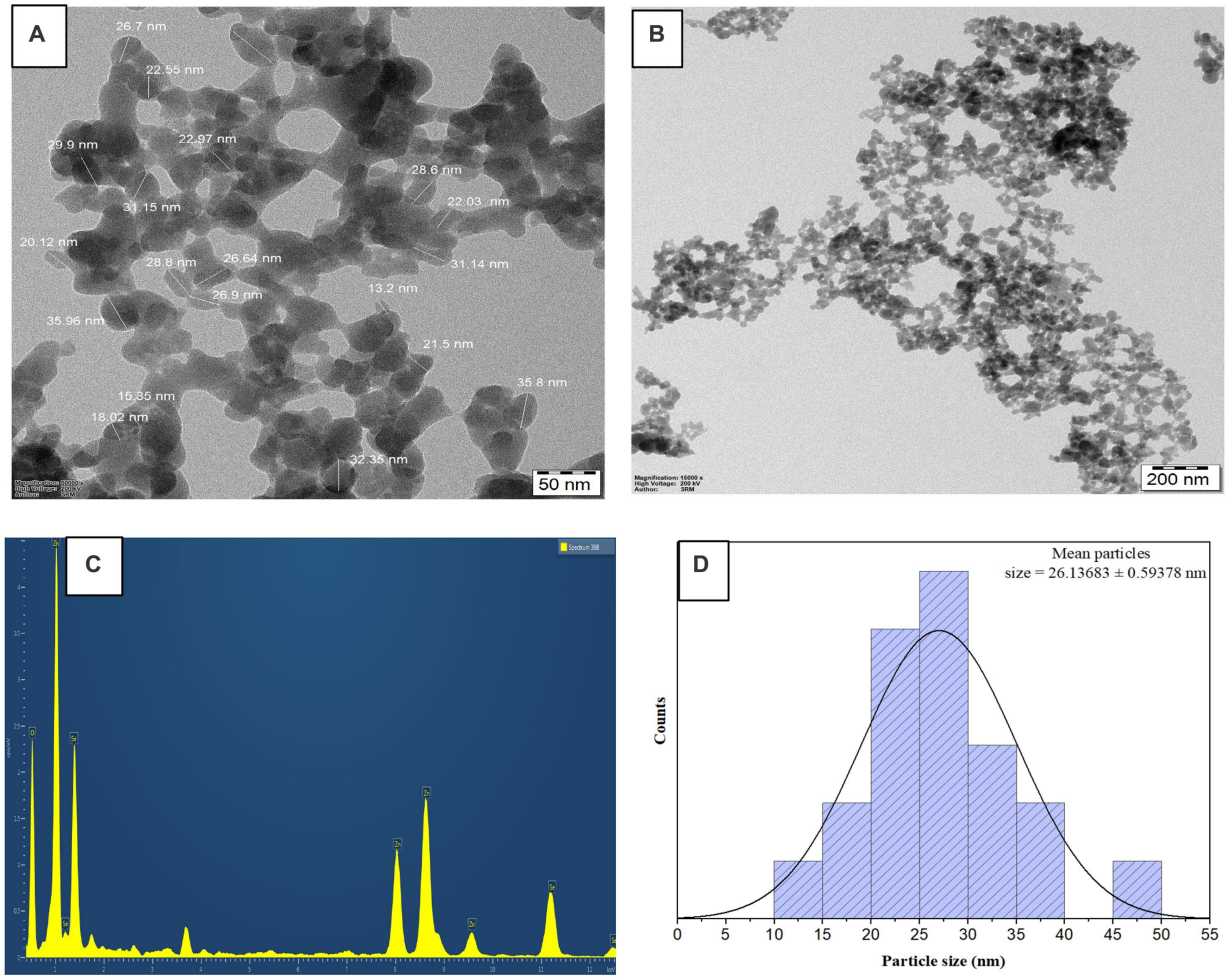
**(A,B)** HRTEM images **(C)** Energy dispersive X-ray spectrum analysis **(D)** Size distribution of bimetallic nanoparticles.

The TEM analysis is to determine the shape, size, dispersion, and surface area of the synthesized bimetallic nanoparticles Se-ZnO NPs (Fouda et al., 2022). The noble nanoparticles are nearly spherical and slightly agglomerated in the obtained TEM image. The average size of the noble nanoparticles was measured by the ImageJ software in the attached Figure 5A. The above-mentioned particle’s average size was observed to be 26.13 ± 0.593 nm. As the Duan et al. (2020) study shows, that confirms EDAX investigation clarified the authenticity of Se-ZnO NPs; elemental analysis was confirmed by a unique confinement peak. A higher rate of selenium (27.15%), zinc (41.11%) and oxygen (31.74%) were detected in the synthesized and categorized selenium and zinc oxide nanoparticles in Figure 5C. The histogram bar graph shown in Figure 5D indicates the nanoparticles dispersion.

### 3.7 Particle size and zeta potential analysis

**Figure 6:**
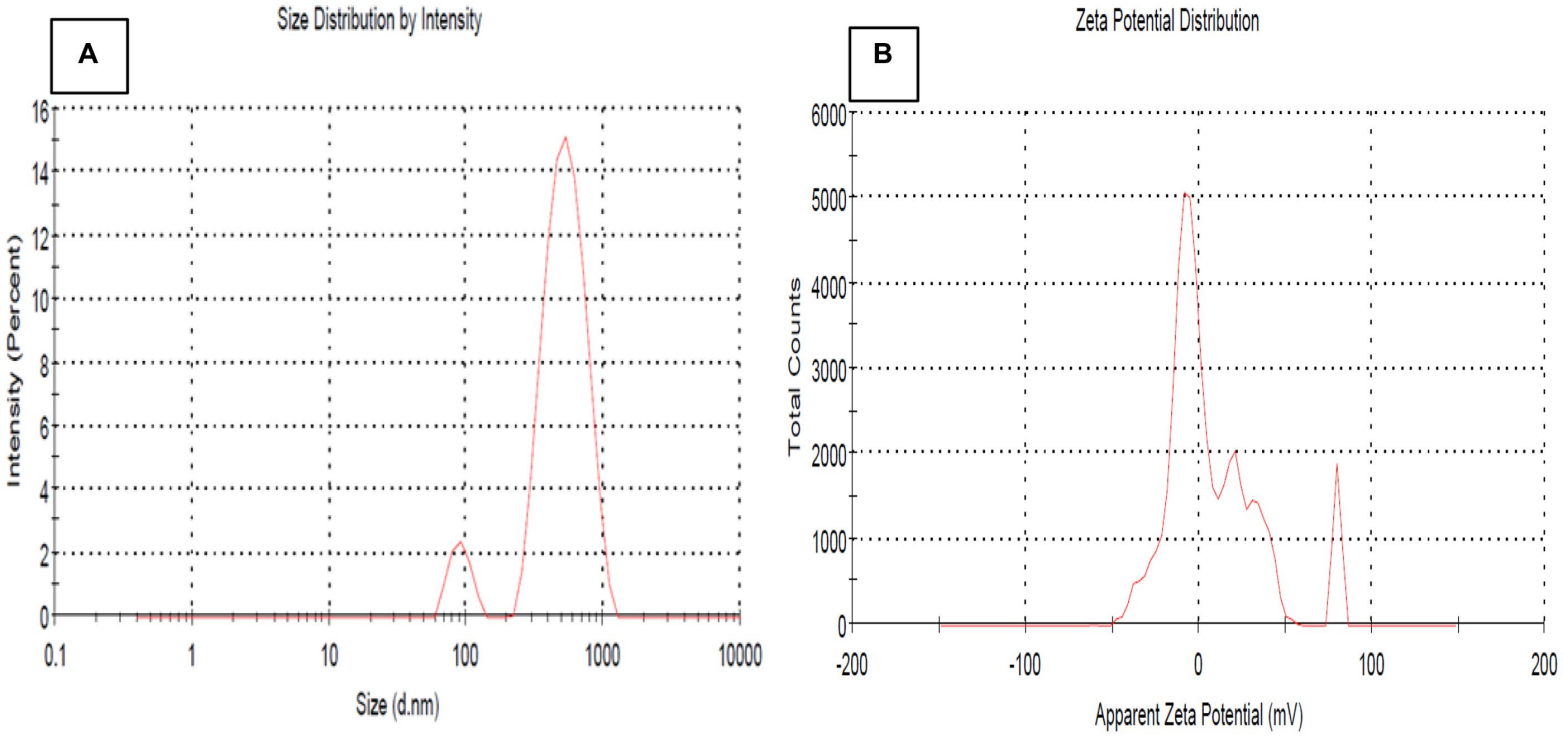
**(A)** Particle size and **(B)** Zeta potential of bimetallic nanoparticles (Se-ZnO NPs).

The particle size of the synthesized nanoparticles was analyzed by DLS (Dynamic Light Scattering) (Chaturvedi et al., 2021). A room temperature and optimal condition were used to calculate the nanoparticle size distribution (Adibian et al., 2022). The bimetallic NPs average value is 398.3 nm, and the PDI was 0.262, as depicted in Figure 6A. Z-average size measures nanoparticle size slightly larger than the actual size due to the hydrodynamic volume measurement in an aqueous state. This is the potential for particle aggregates in the liquid medium. Because of that, Z-average size measures aggregate sizes rather than individual nanoparticle sizes (Sharma et al., 2014; Kapur et al., 2017; Alvi et al., 2021). The synthesized bimetallic nanoparticles are monodispersed, as indicated by their broad size distribution and PDI value of 0.262, which substantiates the monodispersity of Se-ZnO NPs, confirmed by the following study by Umar et al. (2018). Zeta potential is a technique for understanding colloidal suspensions, including nanoparticles and proteins (Hosny et al., 2022). Zeta potential quantifies the electric charge at the interface, affecting particle attraction and repulsion of the synthesis nanomaterials. A study by Mirzaei et al. (2021) indicated that fabricated synthesis of negatively charged nanoparticles with high stability and antioxidant activity is influenced by surrounding biological molecules and zeta potential value changes. The current research study shows that the zeta potential for the bimetallic material is negatively charged at −16.4 mV (Figure 6B). The results indicate the colloidal solution of Se-ZnO NPs, which confirms the stability of the nanoparticles.

### 3.8 Rheological properties

**Figure 7:**
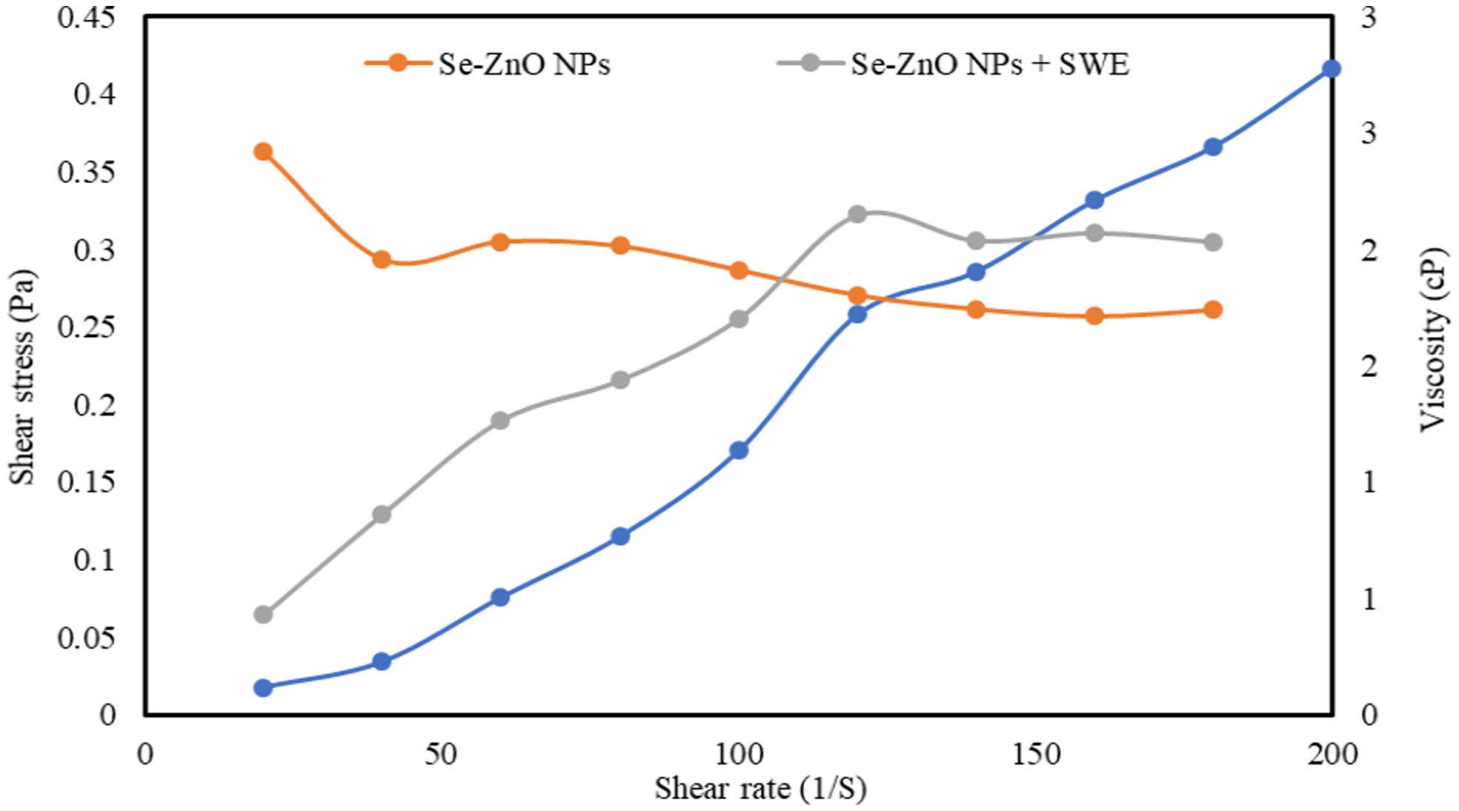
Flow profile properties of Se-ZnO NPs and Se-ZnO NPs + SWE.

In the present study, the experimental result data (shear stress- shear rate and viscosity-shear rate) of Se-ZnO NPs and Se-ZnO NPs + SWE were studied using the rheological Herschel–Bulkley model (Figure 7 and Table 1). The R^2^ value obtained from the flow behavior of the rheological model curve (shear stress- shear rate) indicated a good correlation factor. The shear rate is proportional to shear stress which indicates the linearity and the interaction between the components (Zaim et al., 2020; Rukmanikrishnan et al., 2021). The yield stress (Ʈ_o_), flow behavior index (ƞ) and consistency coefficient (k) were also calculated using the software provided with the rheometer. The Ʈ_o_ values of Se-ZnO NPs + SWE is comparably higher to that of Se-ZnO NPs. The yield stress (Ʈ_o_) defines the macro lattice structure of biopolymeric molecules that helps in determining the interaction ability (Dara et al., 2021). The k value of Se-ZnO NPs + SWE is significantly higher than Se-ZnO NPs, which might be due to the incorporation of nanoparticles. It is known that particle size and shape of the nanoparticle are the most important factors that influence the rheological properties. It is noticed that the viscosity of Se-ZnO NPs decreased with an increase in shear rate, this might be due to the coalescence of nanoparticles (Rukmanikrishnan et al., 2021). The viscosity of Se-ZnO NPs + SWE increased with an increase in shear rate, this indicated that the metallic nanoparticles are embedded in the matrix of biopolymeric molecules of seaweed by an entangled network. The flow behavior indices of Se-ZnO NPs and Se-ZnO NPs + SWE were found to be shear-thinning (ƞ < 1) and shear-thickening (ƞ > 1) fluids. The higher flow behavior index of Se-ZnO NPs + SWE revealed a strong interaction. This reveals the strong network structure and hydrogen interactions between metallic nanoparticles and biopolymeric molecules.

**Table 1:** Herschel-Bulkley model for determining the rheological property indicating the consistency index, flow index and yield stress.

Materials	Consistency index (cP)	Flow index (ƞ)	Yield stress (D/cm^2^)	*R*^2^
Se-ZnO NPs	0.13	0.98	0.05	0.99
Se-ZnO NPs + SWE	3.77	1.48	1.30	0.99

### 3.9 Anti-cancer activity

**Figure 8:**
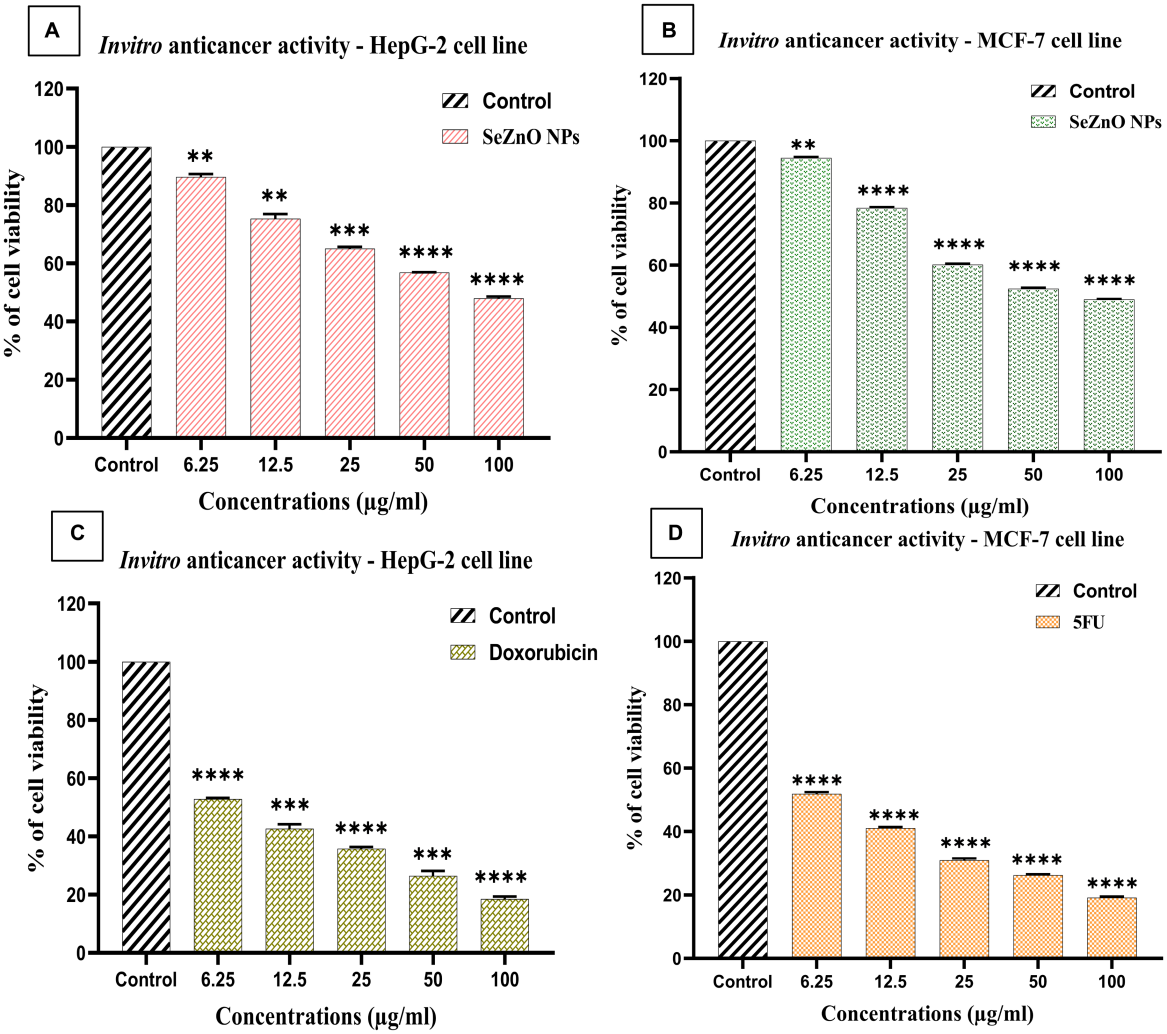
**(A,B)** *in vitro* anti-cancer activity of bimetallic nanoparticles and **(C,D)** standard drugs (5-FU and Doxorubicin) on HepG-2 and MCF-7 human cancer cell lines. **(A)**: MTT assay along with untreated control and variation in different concentrations of Se-ZnO NPs **(A)** MCF-7 cell line against SeZnO NPs; **(B)** HepG-2 cell line against SeZnO NPs.

The IC_50_ values of bimetallic nanoparticles against MCF-7 and HepG-2 cells was found to be 67.9 μg/mL and 79.15 μg/mL, respectively. Whereas, the standard drugs (5FU and Doxorubicin) showed 6.17 μg and 7.48 μg/mL, respectively (Figures 8A,B). The viability of cell line found to decrease with increase in concentration of NPs and standard drugs, which is in coincidence with the study of Valsalam et al. (2019). A study by Yang et al. (2022) stated that the Selenium in combination with doxorubicin has high attenuation of cancer cells. Similarly, the combination of Zinc and 5-FU complexes showed anti-cancer activity against brain tumor cells and breast tumor cells (Hu et al., 2012; Alkış et al., 2021). As mentioned in the study of Saranya et al. (2022), It has been found that the combination of apigenin with Se NPs significantly increases MDA (malondialdehyde) production in cancer cells, suggesting it is a potential therapeutic approach in the fight against breast cancer. There is an interaction between zinc ferrite nanoparticles and cell walls that results in membrane deformation and rupture, the creation of reactive oxygen species (ROS), particle dissolution, and the release of free Zn^2+^ ions, which leads to the production of ROS within cells by mentioned the study of the Sarala et al. (2020). The study demonstrated that synthesized Selenium nanoparticles induce apoptosis via the intrinsic mitochondrial pathway, resulting in dose-dependent upregulation of caspase-9 and decreases in Bcl-2. This leads to increased mitochondrial membrane permeability, apoptosis activators, and caspase-9 activation, ultimately resulting in programmed cell death (Trauzold et al., 2003; Cui et al., 2018). Furthermore, the bimetallic NPs synergistically affected the cancer cell lines MCF-7 and HepG-2 cell lines. The result has been evidence that Se-ZnO NPs cause cytotoxicity in MCF-7 and HepG-2 cells based on the size of the particles and their stability, likewise, mentioned in the studies of Cui et al. (2018), Khan et al. (2021), and Mirzaei et al. (2021).

## 4 Conclusion

In this study, the successful synthesis of Se-ZnO nanoparticles was done, and the initial target of the study was to fabricate a relatively cost-effective and potent alternative system of nanoparticles. The synthesized NPs exhibited the characteristics of both metals and behaved in unison. The extract utilized for the process of green synthesis, i.e., *Padina boergesenii*, a macro alga collected from the Mandapam coastal region of Tamil Nadu; as in previous studies, the extract was prominent in the effective inhibition of multiple cancer cell lines. Based on our study on this and the prospect of the bimetallic NPs, we synthesized the NPs, characterized them, and tested them against HepG2 and MCF-7 cell lines. The BNPs exhibited favorable physical characteristics in terms of size, shape, and overall morphology. Further, the functional groups of the bioactive components from the macro-algae played a significant role in the synthesis as well as increasing the overall efficacy of synthesized BNPs. It is noteworthy to say that the synthesized BNPs exhibited significant IC_50_ values against cancer cell lines. Several studies mentioned that the presence of polyphenolic compounds in higher amounts tends to act as chemo-preventive agents and antioxidants, which might be the important reason for exhibiting anti-cancer activity. The results of the present study suggest that the synthesized NPs by eco- and green technological method could be potential substitute for the development of nutraceutical and therapeutic formulations against synthetic inhibitors of cancer and its related diseases.

## Data availability statement

The raw data supporting the conclusions of this article will be made available by the authors, without undue reservation.

## Ethics statement

Ethical approval was not required for the studies on humans in accordance with the local legislation and institutional requirements because only commercially available established cell lines were used. Ethical approval was not required for the studies on animals in accordance with the local legislation and institutional requirements because only commercially available established cell lines were used.

## Author contributions

BT: Data curation, Methodology, Software, Writing – original draft. YP: Investigation, Methodology, Software, Validation, Writing – original draft. GK: Methodology, Investigation, Data curation, Software, Writing- original draft. PD: Investigation, Software, Validation, Writing – review & editing. SR: Resources, Software, Visualization, Writing – review & editing. SV: Formal analysis, Investigation, Validation, Writing – review & editing. TS: Formal analysis, Investigation, Supervision, Validation, Writing – review & editing. BH: Conceptualization, Project administration, Formal analysis, Validation, Supervision, Writing- review and editing. TD: Conceptualization, Formal analysis, Investigation, Supervision, Writing – review & editing. SS: Funding acquisition, Investigation, Project administration, Supervision, Writing – review & editing.
